# Patient and public involvement in a UK National Institute for Health Research Programme Grant for Applied Research: experiences from the Chronic Headache Education and Self-management Study (CHESS)

**DOI:** 10.1017/S1463423621000670

**Published:** 2021-11-19

**Authors:** Vivien Nichols, Gemma Pearce, David R Ellard, Simon Evans, Kirstie Haywood, Chloe Norman, Rachel Potter, Harbinder Sandhu, Kimberley Stewart, Martin Underwood, Shilpa Patel

**Affiliations:** 1 Warwick Clinical Trials Unit, Warwick Medical School, University of Warwick, Coventry, CV4 7AL, UK; 2 School of Psychological, Social and Behavioural Sciences, Coventry University, Coventry, CV1 5FB, UK; 3 Migraine Action, Leicester, UK; 4 Warwick Research in Nursing, Division of Health Sciences, Warwick Medical School, University of Warwick, Coventry, CV4 7AL, UK; 5 University Hospitals of Coventry and Warwickshire, Coventry, CV2 2DX, UK

**Keywords:** chronic headache, experience, patient and public involvement, primary care, self-management

## Abstract

**Background:**

Patient and public involvement (PPI) plays a crucial role in ensuring research is carried out in conjunction with the people that it will impact upon. In this article, we present our experiences and reflections from working collaboratively with patients and public through the lifetime of an National Institute for Health Research (NIHR) programme grant; the Chronic Headache Education and Self-management Study (CHESS) which took place between 2015 and 2020.

**PPI over the course of CHESS:**

We worked closely with three leading UK migraine charities and a lay advisory group throughout the programme. We followed NIHR standards and used the Guidance for Reporting Involvement of Patients and the Public checklist. We consulted our PPI contacts using a variety of methods depending on the phase of the study and the nature of the request. This included emails, discussions, and face-to-face contact.

PPI members contributed throughout the study in the programme development, in the grant application, ethics documentation, and trial oversight. During the feasibility study; in supporting the development of a classification interview for chronic headache by participating in a headache classification conference, assessing the relevance, and acceptability of patient-reported outcome measures by helping to analyse cognitive interview data, and testing the smartphone application making suggestions on how best to present the summary of data collected for participants. Due to PPI contribution, the content and duration of the study intervention were adapted and a Delphi study with consensus meeting developed a core outcome set for migraine studies.

**Conclusions:**

The involvement of the public and patients in CHESS has allowed us to shape its overall design, intervention development, and establish a core outcome set for future migraine studies. We have reflected on many learning points for the future application of PPI.

## Background

In 2006, the UK government articulated a goal that ‘*patients and public should be involved in all stages of the research process*’ (Research and Development Directorate, 2006). Much progress has been made since then. Patient and public involvement (PPI) in health services research has increased substantially over the past decade. Doing this may help ensure the focus and design of the research is relevant, material is participant friendly, the overall research is feasible and ethical, and finally that implementation is appropriate (Bagley *et al*., 2016). Many funders now expect researchers to involve patients and the public in all aspects of research, from planning, design, delivery of the study, and implementation of findings. The idea being, research should be conducted ‘*with or by*’ the public and not *‘to, about or for’* them (NIHR). This is a shift in the previous assumption that researchers and clinicians were the experts. As the public will be the end users, it makes moral and ethical sense that they should play a key role in the research (Rose, 2014). PPI can enable careful consideration of the relevance, acceptability, and implementation of the research. (Brett *et al*., 2014b; 2014a).

With a growing appreciation of the underlying principles of public and patient involvement, it is timely to reflect on the practical experiences of implementation. Many articles report the use of PPI in their research, but the experience, impact, and outcome of such involvement are not always clear (Brett *et al*., 2014a). The Guidance for Reporting Involvement of Patients and the Public (GRIPP) checklist was the first international guidance for reporting PPI in research (Staniszewska *et al*., 2011). It aims to tackle the inconsistencies in reporting by providing a framework to improve the quality, consistency, and transparency of PPI reporting. The checklist comes in two forms: a long form where PPI is the primary focus of the research and a short form where PPI is not the focus of the research. We report retrospectively on our experiences using the short form GRIPP2 (Staniszewska *et al*., 2017).

In this article, we have tried to be clear where PPI has been used in our work. There are times when we report patient participant involvement which is not classified as PPI (such as cognitive interview participants) however they have been included to add context to the use of PPI around them. This article presents our experiences and reflections from working collaboratively with patients and the public through the lifetime of a NIHR Programme Grant for Applied Research; the Chronic Headache Education and Self-management Study (CHESS - RP-PG-1212-20018) the trial ran from 2015 to 2020. This type of grant allows for multiple work packages and aims: ‘*to deliver research findings that will lead to clear and identifiable patient, service user or carer benefits, typically through promotion of health and wellbeing, prevention of ill health, and optimal disease management (including safety and quality)’ (NIHR).* We hope that our reflections on the inclusion of PPI may help future studies.

## PPI over the course of the CHESS programme

The overall aim of the CHESS programme was to design and test an education and self-management intervention for people with chronic headache (headache on 15 or more days a month, for at least 3 months). Chronic headache types include migraine, tension type, and medication overuse. This body of work started with a feasibility study with four work streams. The main trial was a pragmatic, randomised controlled trial testing the effectiveness and cost effectiveness of a group self-management and education support programme plus usual care for people with chronic headaches, compared to a control of usual care and relaxation (Patel *et al*., 2020). The overall trial design and aspects of our development work are published elsewhere (Patel *et al*., 2019; Potter *et al*., 2019; White *et al*., 2019).

PPI was included at all stages of this programme of work. Table 1 summaries this PPI input.

**Table 1.** Summary of PPI input

Areas of input	Input provided by
**Development work and programme oversight**	
Development of the research idea	Input from three lay members of NICE panel
Development of grant application	Input from three charity partners – The Migraine Trust, Migraine Action, and the National Migraine Centre
Trial oversight	Input from three charity partners – The Migraine Trust, Migraine Action, and the National Migraine Centre
Independent programme steering committee	Two lay representatives recruited with support from the charity partners
Data monitoring committee	One lay representative recruited with support from one charity partner
Ethics application	Input from five lay advisory group members
**Feasibility study**	
Establishing a lay advisory group	Ten of our lay reference group (47) became our lay advisory group
Headache classification conference	Input from seven lay reference group members
Smartphone App development and summary dissemination	Input from four lay advisory group members in the development, and input from four lay advisory group members for the summary dissemination
Intervention development	Three lay representatives recruited with support from the charity partners. Input also from one of our charity partners
Patient reported outcome measures (assessment and analysis)	Input from three lay advisory group members
**Main study**	
Public and media engagement	One lay member and the chief investigator were invited to speak on a national TV show
Interpretation of trial results	Ten members of lay reference group attended a discussion meeting about the main process evaluation and study results
Core outcome set for migraine (COSMIG)	Three PPI research partners who were part of the lay advisory group were involved with the Delphi study

Critical to our PPI activities was the acknowledgment that all members of the team contributed unique expertise to the group, that they were viewed as ‘equal’ within the research team, and that power differentials were to be avoided (Locock *et al*., 2017). However, it was important for all members of the team to be aware of occasions throughout the study when specific questions would require greater input from specific team members. For example, a clinical question may require the specific, technical insight of a neurologist. Alternatively, determining if an outcome measure or intervention was relevant to people with headache would be more readily informed by the perspectives of people living with headache. An awareness of these potential differentials sought to facilitate a mutual respect and understanding between team members. This was further supported by effective team working and a transparency regarding roles. Our PPI members were paid for their time and travel when participating in CHESS activities which are advocated in NIHR involve guidelines. Our charity partners were offered reimbursement for travel.

### Development work and programme oversight

#### Development of research idea

The underpinning research question for CHESS; ‘will an education and self-management programme help people living with chronic headache’, was suggested in the National Institute for Health and Care Excellence (NICE) guidelines on the management of headaches published in September 2012. The three lay members of this group were fully involved in identifying research questions. One lay member of the Guideline Development Group, representing The Migraine Trust, subsequently joined the study team for this research programme as a co-applicant.

#### Grant application

Representatives of the three leading UK migraine charities (Migraine Action^1^, The Migraine Trust and The National Migraine Centre) were co-applicants on the proposal and were given the opportunity to engage in all areas of the research development. The involvement at this stage was from our charity partners rather than specific direct patient involvement. It is difficult on reflection to say how having such patient involvement might have influenced the research question, aims, objectives, or even design. However, our charity partners might not have fully represented the patient voice.

During the first stage of the two stage NIHR programme grant application process, we had proposed a separate work stream evaluating the input of PPI in the programme. We were asked to remove this prior to stage 2 of the full application. PPI activities were still included and costed for in the approved application, but there was no funding for an evaluation.

#### Trial oversight

Oversight for the programme was managed by a programme management group and subsequently a trial management group (TMG), and during the randomised controlled trial (RCT) by an independent programme steering committee (PSC) and a data monitoring committee (DMC). The TMG met monthly in the early phase of the study and subsequently bi-monthly once the RCT was up and running. Our charity partners were members of this programme/TMG and were invited to all the meetings. They had the opportunity to influence interpretation, design, and conduct of the research.

Representatives of each of the three charities were invited to each TMG, however the charities found it difficult to commit their staff to meeting times. Up until the end of 2019, charities were only represented on 63% of possible occasions and not at all in 2020. Meetings in 2020 were less frequent due to being in follow-up and often virtual due to the COVID-19 pandemic. We found that change in senior staff within the charities meant that continuity of input was challenging. It is quite difficult for people without research experience to start inputting part way through a project. On reflection, we had also probably underestimated the training needs for these professional public representatives to engage with the research process.

Our independent PSC was made up of experienced academics, trialists, and two lay representatives. The PSC had an independent Chairperson and met at least once a year to monitor and supervise the progress of the trial. Our PSC had two lay representatives, suggested to us by our charity partners. Up until the end of 2020, we had nine PSC meetings, five face-to-face, and four email summaries. Our lay members were present at all of these.

Our DMC had one lay representative suggested by our charity partner Migraine Action. Up until the end of 2019, we had two face to face and three email reports circulated to the DMC. Our lay member was present at one of the face-to-face meetings and as of January 2020 stepped down from involvement in the CHESS DMC due to change in personal circumstances.

#### Ethics application

We sought lay input for our ethics documentation during the feasibility phase of the study. As this input was required ahead of our lay advisory group being set-up, our charity partners supported us in finding members who would be interested in critically reviewing our patient facing documents including study consent forms and patient information sheets.

During our process of applying for ethical approval for the feasibility study, we had input from five of the original interested lay people on study documentation. This specifically included participant information sheets. They commented on the document and suggested changes. The changes suggested were generally amendments to wording for clarity, formatting, and changes to text size to highlight key points. The overall feedback was positive, and the material was deemed clear and user friendly for a lay audience.

### Feasibility study

The feasibility study had four work streams: (1) Developing and evaluating a telephone headache classification interview, (2) Recruiting people with chronic headache from primary care, (3) Selection of the most appropriate patient-reported outcome measures, and (4) Development and evaluation of an education and self-management support intervention. These are described in detail elsewhere (White *et al*., 2019).

#### Establishing a lay advisory group

We recognised, at the grant application stage, that whilst collaboration with the three charities was important, this might not fully represent the experience of people living with chronic headache and that other lay involvement was needed.

We worked closely with our charity partners who have members from a wide demographic and geographical background, to reach a diverse group of chronic headache patients. Our partners assisted by sending out an email invitation to their contacts (members/clients) inviting them to be a part of CHESS. We also sent out details via a University of Warwick initiative aimed at getting patients and the public involved in research and teaching. From this group, we sought a core group of 8–10 members who lived with chronic headaches. We wanted this group to be able to draw on their experience of living with the condition when supporting the research team. We encouraged participation from both males and females of all ages. No specific skills were required, but members needed to have access to email. We detailed possible input from the group to include:

Box 1. Possible areas of input from a lay advisory group

- plans for participant recruitment, including input into patient information leaflets and screening letters
- design of a headache classification interview and participation at a consensus event
- development and piloting of the intervention where we assess relevance, acceptability and appropriateness of the content of the programme
- choice of clinical and cost-effectiveness outcomes to ensure we captured outcomes that were important to patients
- design of the main trial, including plans for participant recruitment, input into interpretation of trial findings, and subsequent dissemination

Following the distribution of our call for PPI members by our charity partners and the university PPI initiative, we received 47 expressions of interest. We named this pool of people our lay reference group. We emailed those that responded, to ask them to complete a questionnaire. They were asked for availability for meeting time preferences, age group, gender, first part of their postcode and headache type (migraine, tension type, medication overuse or other). We received 22 completed responses. Of this group, 10 people responded to an email sent in February 2016 inviting them to submit ideas for group rules of engagement and a group name. As we had planned for a group of 8–10, there was no need for short listing of members.

This group of 10 agreed on a name, the CHESS Lay Advisory Group and the following rules of engagement:

Box 2. Lay advisory group rules of engagement

- To treat all CHESS-related documents/correspondence as confidential
- To reply to correspondence in a timely manner
- To agree or decline projects in a timely manner - based on input required, time to complete and fees payable
- Group members may be identified to each other (voluntary), so that email contact/discussions could take place
- To inform the CHESS team about any change of contact details
- Inform of email change and supply a list of known major holidays or non-available dates to the CHESS team, so that a chart can be drawn up and managed
- Consider cost-effective communication methods: conference call/face to face meetings/doodle polls
- To complete invoices in a timely manner
- To avoid unnecessary jargon
- Discussions and comments should focus on constructive comments and criticisms, keeping an unbiased viewpoint
- To treat all members with due respect at all times: this includes recognising that members may have different opinions and perspectives on issues; and that group members have different needs and requirements
- To work together to encourage and involve participation within the group and to be supportive of other members
- To regularly review the activity of the group and introduce improvements where necessary

Our final group of 10 people was from a wide geographical area. The group comprised of four females with migraine, two females with migraine and tension type headache (TTH), two females with migraine, TTH and medication overuse headache (MOH), one female with migraine and MOH and one male with migraine.

We built a relationship with this group primarily using email due to their dispersed geographical locations. Email contact was maintained with several of the research team members to build relationships, which forms a key part of PPI (Wilson *et al*., 2015). The team also provided a newsletter to inform members of the study developments.

Due to the nature of the programme grant, there were times where specific input was required from our PPI members and at such times contact was made via email.

The input from the lay advisory group was fed back to the wider CHESS team via members of the research team who had been directly consulting with this group.

#### Headache classification conference

We held a headache classification conference to reach consensus on key questions to inform the content of a telephone classification tool for use by non-specialists to classify the common headache types. Full details of the classification tool development and validation can be found elsewhere (Potter *et al*., 2019). We invited our lay reference group to contribute to the day to ensure the key questions generated were important to both health professionals and people with chronic headache.

We invited the 22 people from our lay reference group, detailed above, to attend the day. Seven lay people attended on the day and were randomly allocated to one of four groups alongside neurologists, general practitioners, nurses, and allied health professionals. We had planned to have two lay members in each group, but one participant was unable to attend on the day. Experienced facilitators had previously discussed potential power imbalances within groups as part of their preparation and actively encouraged involvement from all members of the group. The study team used the output from the conference to develop a logic model that underpins the telephone headache classification interview.

#### Electronic data capture and summary documentation

As part of the CHESS trial, we wanted to develop and use a smartphone application to collect information on frequency, severity, and duration of headaches on a weekly basis for 6 months followed by a monthly basis for a further six months. We invited our lay advisory group as consultants to provide feedback on the user experience and acceptability of the application.

In developing this application, we had feedback from the group, who helped to refine the questions to be used in the application.

Specifically, the advisory group were asked to assess the ease of downloading and installing the application, the wording of the questions, the response options, instructions on completion, time taken to complete, and other general comments.

The research team planned to provide feedback to trial participants summarising their electronic diary data at the end of the data collection period. A draft of the summary was developed by the research team and circulated to the lay advisory group. Two versions were circulated, and the group was asked to comment on which document they preferred, if the content made sense and any recommended changes to improve the documents. Four people responded, their feedback allowed us to revise the document and select the one that was deemed more user friendly and clear.

#### Intervention design and development

We worked with migraine action to send letters to 100 of its members inviting them to discuss their headaches and what treatments they had tried. The aim was to use this information to help inform the intervention development. We received 21 responses from the invitations sent out by migraine action. Seven of these people lived with chronic headache meeting our inclusion criteria as detailed elsewhere (White *et al*., 2019; Patel *et al*., 2020) and took part in a face-to-face, semi-structured interview. Qualitative thematic analysis of their responses gave us some ideas on what they thought should be included in an education and self-management group intervention. They also provided some practical ideas about the arrangements of such groups.

Following this on the 9th November 2015 at the Royal College of General Practitioners we held an intervention design day meeting. This meeting was attended by 18 members including academics, clinicians, behaviour change and self-management experts, our charity partners, and three PPI members suggested by our charity partners. The aim of the meeting was to review results from; a series of systematic reviews (Nichols *et al*., 2017; Probyn *et al*., 2017a; 2017b), the consensus conference, and the qualitative data gathered to identify the key learning points and factors that should inform the development and design of the CHESS intervention. The day was chaired by one of our charity partners (SE) and presentations were followed by facilitated discussions to identify the core messages coming out from the different streams of work. Specific consideration was made around what the intervention and the control arms should look like, content, structure, delivery, and practicalities. Consideration over the level of ongoing support, written material, and other resources were also discussed.

Through PPI, we gained a good insight into the challenges of managing chronic headaches and the practicalities of running a trial in this population. A key insight from this meeting was the potential merit of involving participants’ partners and families in the group intervention sessions. This was recognising that many people living with chronic headaches find it difficult to explain how it affects them to their partners and families. This was something the lay people at the intervention day flagged up, it was discussed at that meeting and it was considered that there would be issues around; time, logistics, confidentiality, and that not all would have someone to bring. To address this concern, we produced a short (25 min) video aimed at both participants and their families to include as part of the resources provided during the CHESS intervention.

Following the review of the evidence base and detailed discussions, a summary document was created and circulated to the attendees. The document aimed to summarise the detailed discussion and key decisions made on the day (discussions were captured on the day via detailed notes from the study trial manager).

The structure, content, and design of our intervention were influenced by findings from our systematic reviews and PPI input. When designing the intervention, there had been all round agreement that the intervention should be facilitated by a nurse and a lay person who has chronic headaches. The rationale for this being they can bring their own personal experience to the group-based intervention (Taylor *et al*., 2016a; 2016b). The practical application of this was more challenging. From our experience, we struggled to recruit enough lay people with chronic headaches willing, and able, to deliver the intervention for us. Some of this might have attributed to the ad hoc nature of the work, but based on those we did recruit and train, it was the uncertain nature of their headaches that caused them anxiety and therefore restricting their willingness to volunteer as lay facilitators. After much consideration, and with the support of the PSC, the trial management team decided to replace the lay person with an allied health professional.

The original intervention design, after the input of our lay advisors was a two-day back-to-back self-management group programme followed by a one-to-one consultation with the nurse and finally a half day group follow-up. The intervention was designed and structured in this manner, but during our pilot study, the feedback from participants and facilitators suggested participants preferred a shorter course due to work commitments. Having two days back-to-back was also challenging for participants to focus, and they preferred a gap between the two sessions. As a result of this feedback, we revised the intervention to two days plus a one-to-one consultation. We also split day one and two with a one-week gap in between.

#### Patient reported outcome measures (PROMs)

PROM selection was informed by a systematic review of the psychometric properties of available measures for people with chronic headache (Haywood *et al*., 2018). However, no studies explored the relevance of these measures to patients. During the feasibility phase of the trial, we interviewed participants to better understand the relevance and acceptability of our proposed outcome measures. Cognitive interviews were conducted by an experienced qualitative researcher who was also part of the independent process evaluation team of the study. We interviewed fourteen participants from the feasibility study using a semi-structured schedule. We sought their views of the relevance, comprehensiveness, clarity, and acceptability of two headache-specific measures and two generic measures of health status used in the study. Three members of the CHESS lay advisory group contributed to the analysis of interview findings, also actively participating in an analysis day. The aims of the analysis, using framework analysis (Ritchie and Spencer, 2002) and cross-case comparison, were stated before the day with instructions for them to complete the measures for familiarity. We gave background information about salient research methods and how their views would contribute to the analysis. The results of the interviews were then explored with two members of the CHESS team and recommendations were made. The PPI partners contributed to an associated publication (Haywood *et al*., 2021).

### Main study

#### Public and media engagement

The University published a press release on 29th of January 2018 which attracted attention from local and national bodies. This was picked up by the BBC Victoria Derbyshire show, where the study was represented by one of our lay members and the chief investigator. Our lay member had the opportunity to speak about their experience of living with chronic headaches and the impact this has. The programme generated public interest in the trial and the central study team received calls from interested individuals, some of whom went on to participate in the study. Feedback from our PPI member indicates that this was a key time in the research process they had felt involved in the team rather than on the periphery, which was very important to them.

#### Trial results

We sent emails to our original lay reference group inviting them to attend a two-hour online meeting to help us to interpret the main trial results. Ten people agreed on a specific date and time. Two members of the CHESS process evaluation team presented the main results from firstly the process evaluation followed by a short summary of the main trial results. These results were discussed, and the group was asked about; their thoughts on the results, how these might be received and where the research should go from here. The trial results are not available at this time but this PPI input will be included in the main paper and report. Lay members will also be asked to help with the report lay summary.

#### Core outcome set for migraine (COSMIG)

The aim was to develop a core outcome set for chronic and episodic migraine (COSMIG) ensuring that the perspectives of both people with migraine and health professionals influenced recommendations. Three members of the CHESS lay advisory group joined the COSMIG core team as PPI research partners. They were active members of all COSMIG meetings, contributing to the co-production of a three-stage, international e-Delphi survey and subsequent data interpretation, co-facilitation of a multi-stakeholder consensus meeting, and co-authorship of associated publications (Haywood *et al.,* in press).

The PPI partners informed the use of relevant, accessible, and jargon-free language. This helped with advertising the study, developing the Delphi questionnaire, and information packs for consensus meeting participants. The use of plain English supported meaningful patient participation throughout. The relative success of this was evidenced by the high completion rates (80%) and high retention rates (>70%) throughout the e-Delphi study. Their contribution to data analysis at each stage ensured that the perspective of patients was not lost. Patient and health professional data was analysed both separately and combined to highlight differences in important outcomes.

The Delphi process resulted in the short listing of seven aspects of health (pain, overall health, usual activities, cognitive function, adverse effects, associated symptoms, and self-management). These were considered further by participants in a face-to-face consensus meeting. Seven health professionals and seven patients who had completed all three stages in the Delphi study participated. The meeting consisted of both small and large group discussions, followed by voting on the core domains and outcome measures. These discussions were facilitated by members of the core COSMIG team, including two of our three PPI partners. Our PPI partners ensured that the documentation provided to all participants was written in plain English and user-friendly. They also supported patient participants during the group discussions and voting processes. As members of the core team, the PPI partners did not participate in the voting process (Haywood *et al.,* in press).

## Discussion and reflection

Comprehensive reporting of PPI in health research publications is poor (Price *et al*., 2018). In 2020, the National Institute for Health Research (NIHR) released new UK Standards for Public Involvement aimed to improve the quality and consistency of public involvement in research (NIHR, 2019). A framework for what good public involvement should look like includes the following six standards: communication, working together, inclusion opportunities, impact, governance, and supporting and learning. These are useful tools moving forwards as we reflect on how PPI has helped shape various aspects of the CHESS programme including the intervention and classification tool. A discussion of our experiences and the impact of PPI throughout the CHESS initiative is structured around four key concepts important to understanding the effectiveness of PPI: reach, refinement and improvement, relevance, and relationships (Staniszewska *et al*., 2018).

*Reach* is the extent to which individuals and communities engage, participate, and are involved in research to ensure diversity, and by creating environments that allow people to feel respected.

Early in the programme grant, we became aware of the need to increase the reach of our PPI involvement beyond our charity partners. Our approach was to compile a lay reference group and from that a core lay advisory group which we could draw upon at key points in the research and development process. Assisted by our charity partners, we were able to approach a diverse population, from a wide geographical area, of different ages and with different types of chronic headache. Our approach meant we could not engage with those who had no email access.

*Refinement and improvement* refer to a need to evaluate how PPI is adding value to research excellence. Without doubt, PPI added value to the development and content of the CHESS education and self-management intervention. For example, lay representation at the intervention development day highlighted the need for information for family members to help them to understand the impact of headache. Although not evaluated directly, the contribution of PPI partners ensured the crafting of a relevant and accessible questionnaire that had great resonance with patient participants. Throughout the e-Delphi process, there was high patient participant response and retention rates, thus ensuring the patients voice remained strong. This result ensured that the recommended outcomes have relevance to all stakeholders.

*Relevance* is the extent to which public priorities for research are reflected in funding activities; this is important because public money should be spent on research that is relevant to patients and public. Our charity partners contributed to the programme grant application, but input from people with headache at this early development stage may have strengthened the patient voice in the research.

*Relationship* refers to the importance of building and maintaining relationships that allow for equal power sharing and where roles and responsibilities are clearly defined. We established the CHESS lay advisory group at the start of the programme of work, developing and agreeing on rules of engagement that valued the contribution of different viewpoints and encouraged mutual respect and support. Additionally, PPI members were recognised for their input, acknowledged as contributors or co-authors on publications, and were compensated for their time and where relevant travel. One of our authors as PPI collaborator has been invaluable in helping to shape this paper as well as contributing to multiple work streams throughout the CHESS study. Most importantly, their presence with the chief investigator on a high-profile television show not only promoted research into chronic headache but also hopefully demonstrated that the patient voice is valued in research.

On reflection, our thinking has developed substantially since we wrote and started the NIHR programme grant. The first application for funding was submitted in June 2012. If we were doing this again, there are ways we could improve the quality of PPI to large programmes of work of this nature. Our key learning points are as follows:

Box 3. Key Learning Points

1. There have been missed opportunities in our work due to the lack of PPI review and evaluation. We have been able to detail and document what was done and the changes this created but we have failed to capture the experiences and feedback of those involved. For future studies, we would build in this evaluation to ensure we have robust data. Other studies have recognised the increased time and funding needed for this work (Domecq *et al*., 2014) and the reporting of PPI activities (Fergusson *et al*., 2018).
2. A designated team member to be the PPI contact could have improved engagement. This would allow for more dedicated time to develop and sustain a relationship which is a significant determinant of success (Howe *et al*., 2017).
3. As part of this process, more regular correspondence to update the group on developments as well as provision of timely feedback on how their input has helped to shape the study would be beneficial. We had intended to send out a regular newsletter however due to time constraints the team only managed to circulate one newsletter. We are aware that reciprocity and feedback have been regarded as essential components for effective PPI but observed as often missing (Mathie *et al*., 2018).
4. The feedback of the lay advisory group was generally relayed to the research team by those involved in gathering this feedback, in the future, it would be valuable to obtain the feedback directly from the group to avoid any potential mis/reinterpretations.

We should consider the same training in research process for charity representatives as we, now, provide for lay representatives. Although we did not provide such dedicated training for the role for lay members of TMGs at the time this study started, the Warwick Clinical Trials Unit have subsequently introduced a training programme for PPI which in the future we would encourage all members to attend. https://warwick.ac.uk/fac/sci/med/research/ctu/ppitraining/.

Although our charity partners gave important input, and they will be very important when it comes to dissemination of the results, it might have been better to have had lay people with chronic headaches as members of the TMG as well as the lay advisory group. Likewise, specific lay input into the grant development might have also provided a different perspective and may have provided better continuity of lay input throughout the programme of work.

We have asked members of our lay reference group to help us to interpret the main trial results and assist in developing a lay written summary of the findings. The input from the group will help us to interpret, publish and disseminate our findings with the chronic headache population in mind. This will help us to position our results and produce future research ideas. The trial results will be sent to those participants who have requested the study findings and will be published on a study-specific website. Our charity partners will also be involved with feedback to the organisations they represent.

## Conclusions

PPI has been an important component of shaping the overall design and development of the CHESS trial. This has included thoughts around who should deliver the intervention, the length and structure of the programme and what outcomes are important to people living with chronic headache. Through reflection of our experiences, we have identified key strategies to ensure that future involvement of PPI in research is efficient, rewarding, and supportive for all those involved. We are extremely grateful for the time commitment and enthusiasm from all PPI members involved in the CHESS study.

## Data Availability

The datasets used and/or analysed during the current study are available from the corresponding author on reasonable request.

## Footnotes

1 In 2018 Migraine Action merged with the Migraine Trust - https://www.migrainetrust.org/
